# Southern expansion of the invasive ant *Wasmannia auropunctata* within its native range and its relation with clonality and human activity

**DOI:** 10.1371/journal.pone.0206602

**Published:** 2018-11-21

**Authors:** Lucila Chifflet, Noelia Verónica Guzmán, Olivier Rey, Viviana Andrea Confalonieri, Luis Alberto Calcaterra

**Affiliations:** 1 Departamento de Ecología, Genética y Evolución, Facultad de Ciencias Exactas y Naturales, Universidad de Buenos Aires, IEGEBA (CONICET-UBA), Buenos Aires, Argentina; 2 Laboratoire of Interactions Host-Parasites-Environment, Université de Perpignan, CNRS, IFREMER, Université de Montpellier, Perpignan, France; 3 Fundación para el Estudio de Especies Invasivas, Hurlingham, Buenos Aires, Argentina; 4 Consejo Nacional de Investigaciones Científicas y Tecnológicas (CONICET), Buenos Aires, Argentina; Universidade de São Paulo, BRAZIL

## Abstract

The little fire ant *Wasmannia auropunctata*, native to the Neotropics, has become a serious pest worldwide over the past 100 years. It was originally distributed from Mexico to northern Argentina and new evidence suggests a recent southern range expansion during the last 60 years reaching central Argentina. This supercolonial ant species has a polymorphic reproductive system. Some populations, mostly found in undisturbed natural environments, are characterised by a classical sexual haplodiploid reproductive system. In other populations, which mainly occur in human-modified habitats, diploid queens and haploid males are produced clonally while workers are produced sexually. Here we studied the association between the recent southern range expansion of *W*. *auropunctata* in relation to human activity and clonality. We carried out an extensive survey within the southern limit of the species’ native distribution and characterised the type of habitat where populations were found. Moreover, we genetically determined the type of reproductive system in 35 populations by genotyping at 12 microsatellite loci a total of 191 reproductive individuals (i.e. queens and/or males). Clonality was the most common reproductive system, occurring in 31 out of 35 populations analysed. All the populations found in the recently colonised area in central Argentina were clonal and established in human-modified habitats, suggesting that clonality together with human activity might have facilitated the southwards expansion of *W*. *auropunctata*.

## Introduction

Earth's biota has experienced profound changes as a result of biological invasions [1], which are unlikely to be exclusively associated with human activity based on the dynamic nature of species' ranges [2,3]. However, over the past 200 years there have been large-scale range shifts attributed to anthropogenic influence [2, 4]. The expansion of global trade and transport has led to a marked increase in the geographic scope, frequency and number of species involved in biological invasions [5, 6]. Currently, our planet is suffering from biological invasions at a much higher rate in comparison to prehistoric times [4]. Thus, the accidental introduction of exotic species is an ongoing serious problem that results in unprecedented loss of native species and biotic homogenisation worldwide [7–10].

At a regional scale, human alteration of the environment is also responsible for species extinction and biotic homogenisation [11]. Urbanisation and agriculture create homogeneous landscapes forcing native species to adapt to novel environmental conditions, which are often radically different from the surrounding undisturbed habitat [12]. Under such scenario, many ecological specialists would become locally extinct and would be replaced by a few generalist species able to tolerate the new habitats promoted by humans [13]. Adaptation to human-altered habitats could contribute to the evolution of invasive populations (i.e. populations able to invade areas outside their native range), a scenario recently called ‘Anthropogenically Induced Adaptation to Invade’ (AIAI) [14]. Humans modify habitats in similar ways worldwide, allowing propagules from populations adapted to human-modified environments in the native range to establish successfully within similarly human-modified habitats in the novel range [14]. This scenario reinforces the need to focus invasion biology research not only on the introduced range but also on the native range, where key evolutionary processes promoting biological invasions may occur [14].

The little fire ant, *Wasmannia auropunctata* (Roger, 1863) (Hymenoptera: Formicidae), is an example of a worldwide invader that seems to conform to the AIAI scenario [14]. It is considered one of the 100 worst invasive alien species of the world by the International Union for the Conservation of Nature (IUCN) because of its negative impact on natural ecosystems, agriculture and human health [15].This ant is native to the Neotropics, from where it has spread throughout the world over the past 100 years as a consequence of the expansion of human activities [16]. It is mainly distributed in tropical regions and has been recently reported from Israel in the Mediterranean region, with temperate climate and harsher weather conditions than the tropics (i.e. minimal annual temperatures as low as 6°C and 5–12 consecutive rainless months) [17].

The remarkable success of *W*. *auropunctata* may be attributed to characteristics shared with other invasive ant species (e.g. generalist food and nesting habits, superficial nests, high mobility of the colony, polygyny, low intraspecific and high interspecific aggression, small size, and use of extrafloral nectaries and honeydew from aphids, mealybugs and scale insects) [16]. In addition, its invasive success is highly associated with its particular reproductive system. Some populations of this species (hereafter called sexual populations), have a classical haplodiploid reproductive system in which diploid females (i.e. queens and workers), are produced via sexual reproduction, whereas haploid males develop from unfertilised eggs through arrhenotokous parthenogenesis [18]. In other populations (hereafter called clonal populations), both queens and males are clonal [19] but differ in their mode of reproduction. Diploid queens reproduce through automictic thelytokous parthenogenesis, a system showing strongly reduced recombination rates [20] by which new reproductive females (gynes) are genetically identical to their mother (bearing rare mutations and recombination events). On the other hand, males are produced by androgenesis (i.e. production of haploid male offspring from maternal eggs having strict paternal genome inheritance), whereas workers are sterile and are produced by sexual reproduction as in sexual populations [19]. The clonal reproductive system requires both sexes for worker production and for a high hatching rate of eggs to sustain numerous and functional colonies [21]. *Wasmannia auropunctata* colonies usually consist of several nearby nests (hereafter called clusters) led by one or more reproductive queens. Colonies spread by budding, where one or more queens begin a new colony close to the parental one aided by workers [19].

In the species’ native range, the polymorphic reproductive system has been recorded only in northeastern South American populations (French Guiana and Bahia in Brazil) [18, 22]. Interestingly, the reproductive system of a population (i.e. clonal versus sexual) is strongly associated with the type of habitat. Sexual populations are usually not numerically dominant (i.e. with low density of workers, brood, queens and nests), and establish mostly in natural environments with little or no human disturbance (e.g. primary or secondary forests), whereas clonal populations are usually numerically dominant (i.e. with high density of workers, brood, queens and nests) and colonise human-modified habitats [22]. Moreover, the fact that the latter display the same ecological traits and select such habitat type in both native and introduced ranges [23, 24], strongly suggests that invasive populations outside their native range originate from populations established in marginal habitats within their native range [25], being an example of the AIAI process [14].

The last surveys carried out in Argentina showed that *W*. *auropunctata* is widely distributed, being much more common in human-disturbed than in natural habitats [26]. Interestingly, the native range of *W*. *auropunctata* might have spread southwards over the past 60 years [26, 27], as its southernmost distribution limit has recently been reported to be 34°51'S [26] in central Argentina, two degrees of latitude higher than those recorded previously [16], near the confluence of the Paraná - Caracarañá Rivers (~32°30'S) [28]. Excitingly, one of the populations that recently colonised central Argentina (Zárate, Buenos Aires province) would have acted as the source of the invasive *W*. *auropunctata* in Israel [29]. Hence, the characterisation of the populations within central Argentina may help identify key potential factors facilitating the southward expansion to areas adjacent to its native range that could explain the invasion success in harsh environments (i.e. Israel). In this context, the aim of this study is to assess a possible relationship between the recent southward expansion of *W*. *auropunctata* in Argentina and both human activity and clonality.

## Materials and methods

### Sampled populations

Field surveys were conducted in the southern half of the native distribution area of *W*. *auropunctata* in Argentina and neighbour countries (i.e. Uruguay, Brazil, Paraguay and Bolivia). Permissions to collect samples were issued by Administración de Parques Nacionales and by the authorities of each province in Argentina, Ministerio do Meio Ambiente, Brazil, and Museo de Historia Natural Noel Kempff, Bolivia. Uruguay and Paraguay did not require specific permissions to collect not endangered or protected species. We examined environments with different levels of human disturbance for the presence of *W*. *auropunctata*, ranging from primary and secondary forests to highly human-modified environments (i.e. urban areas, plantations and roadsides). On each sampling site we looked for nests and collected 10–30 workers, 2–10 queens/gynes and 2–10 males per nest. We recorded geographic coordinates and other relevant data for each sampling site (e.g. type of habitat) (S1 Table).

Because the abundance of *W*. *auropunctata* depends on the type of environment [26, 30], the sampling techniques differed between highly human-disturbed and undisturbed or slightly disturbed habitats. The first surveys were carried out between October 2008 and January 2009 (during most of spring) in natural forests at different disturbance levels: 1) Paranaense Forest (slightly modified Atlantic semi-evergreen rain forest) in northeastern Argentina, mainly in Misiones province), 2) Yungas Forest (a slightly to moderately modified low mountain forest with heavy seasonal rains) in northwestern Argentina), and 3) Chaco/Espinal Forest (a more dry and open forest which is currently highly fragmented and disturbed) [31]. Ants were sampled from a total of 30 sites (9 in Chaco/Espinal, 10 in Yungas and 11 in Paranaense Forests; S2 Table) using a combination of two collection methods (sieving and baiting) at sampling points located every ~10 m along a linear transect. Sample size at each site varied between methods depending on the presence, coverage, and depth of the leaf-litter layer. Overall, a total of 634 samples of leaf-litter and above-ground ants were obtained using sieves (5.8 ± 0.4 samples per site) and baits (13.7 ± 0.6 samples per site).

A sieve was used to determine the presence and abundance of *W*. *auropunctata* and other ant species in the leaf-litter. At each sampling site, all leaf-litter (leaf mold, rotten wood) inside a 0.25-m^2^ quadrat was collected and sieved through a 1x1 cm mesh sieve above a white plastic tray (12x45x55 cm). Quadrats were located at >50 m away from the edge of the forest. Ants were collected from the tray with forceps and preserved in 96% ethanol for taxonomic identification. A total of 146 quadrats were sampled: 55 in Yungas, 46 in Paranaense and 45 in Chaco/Espinal.

Baits were used to assess the presence and abundance of *W*. *auropunctata* and other terrestrial ants and to lure workers to detect their nests. Baits consisting of 1–2 g of canned tuna were placed on a 5x5 cm white paper card for 30–45 min at each sampling site. All ants attracted were stored in 96% ethanol for further identification. Ants were attracted to a total of 488 baits: 133 in Yungas, 183 in Paranaense and 172 in Chaco/Espinal.

Ants collected with both methods were identified to species or morphospecies under a dissecting scope with available keys [32–35] by L.A.C. and Fabiana Cuezzo of the Instituto-Fundación Miguel Lillo (IFML) in Tucumán province, Argentina. Voucher specimens were deposited at the entomological collections of the IFML and the Fundación para el Estudio de Especies Invasivas, Argentina.

Between December 2009 and October 2012, sampling consisted of searching for nests in human-disturbed environments (e.g. cities, roadsides and plantations), and in a few undisturbed and slightly disturbed habitats that had not been sampled before. The sampling was carried out along main routes and dirt roads (S1 Fig), choosing sites that meet the nesting requirements of the species (i.e. moist habitats with stones, tree barks, dead wood, leaf litter, etc.) [16]. Samples were collected manually for about 45 min by direct searching under barks, logs, stones and sidewalk tiles in cities. Specimens were preserved in 96% ethanol and deposited at the Laboratory of Molecular Phylogenies and Phylogeography (EGE-FCEN-UBA, Argentina). The collected material was examined under a stereoscopic microscope to confirm the species identity. Overall, using all the collecting methods, we sampled *W*. *auropunctata* from a total of 93 locations (S1 Table).

### Reproductive system characterisation

We analysed the reproductive system of the populations where reproductive individuals (queens, gynes and/or males) were found (35/93) (S1 Table). Of these, 26 corresponded to highly human-modified habitats (i.e. cities, roadsides or plantations) and 9 to undisturbed or slightly modified habitats (primary and secondary forests and naturally inundated floodplains) (S1 Table). We genotyped 191 reproductive individuals (182 reproductive females and 9 males) at 12 microsatellite loci as described in [36]. PCR products were separated on an ABI 3130 DNA sequencer (Applied Biosystems). Fragments were analysed using GeneMapper v.3.7 (Applied Biosystems).

### Data analysis

We characterised the reproductive system of each population by comparing among individual multilocus genotypes visually (following [18]) and using GenClone v.2.0 [37]. The number of multilocus genotypes present in the whole sample was identified and the different individuals were sorted into groups with the same genotype (exactly identical at all loci). A population was defined as clonal when comprising female or male genotypes identical at all microsatellite markers [36].

The program mentioned above was used to identify individuals from a same clonal lineage by considering possible mutation and/or recombination events that they would have experienced [38]. This should be kept in mind to avoid overestimation of the true clonal diversity of a population or species [38, 39]. To this end, we constructed a matrix of allele distances between all pairs of genotypes in the sample and then represented these values by a frequency histogram. Populations of a species having a mixed reproductive system (i.e. clonal and sexual) are expected to exhibit a bimodal frequency curve characterised by a smaller peak corresponding to individuals with few allelic differences that belong to the same clonal lineage (associated with a single sexual reproduction event) and a larger peak corresponding to individuals with many allelic differences, considered as belonging to different lineages (each one arising from a distinct sexual reproduction event) [38]. To establish the maximum allelic difference to assign two individuals to the same clonal lineage, we used a sub-dataset of our samples (individuals from localities in the Paraná River Delta region, i.e. Buenos Aires, Zárate, Camping Las Tejas, San Pedro, San Nicolás and Rosario). We found a distinct peak at small genetic distances (1–3 alleles across the 12 diploid loci) indicating samples that derived from the same sexual reproduction event and only differed due to mutations or recombination events during asexual reproduction. Accordingly, we grouped all individuals sharing 3 or less allelic differences into the same clonal lineage [38, 40].

To study the genetic relationships between different populations we constructed a Neighbour-joining dendrogram [41] using the genetic distance between individuals with MEGA v.6 [42].

## Results

### Habitat type

Of the 93 sampling sites where *W*. *auropunctata* was found, 77% (72 sites) corresponded to highly human-modified habitats (i.e. roadsides, plantations and urban environments), and 23% (21 sites) to undisturbed (i.e. primary forests) or slightly disturbed habitats (i.e. secondary forests and floodplains) (S1 Table). In total, we recorded 253 nests grouped into 98 clusters.

The first surveys carried out in undisturbed habitats using sieves and baits showed that *W*. *auropunctata* is not abundant in natural forests in Argentina. It occurred at 37% (11/30) of the collecting sites, in only 6% (36/634) of the leaf-litter and bait samples (S2 Table). It was three times more abundant in leaf-litter (18/146) than in bait (17/488) samples (S2 Table), with differences among the three forest types. In the Chaco/Espinal it occurred at 7 of 9 sites and was found only in 11% (25/217) of the samples. In the Yungas it occurred at 1 of 11 sites and in 3% (5/188) of the samples, and in the Paranaense it was present at 3 of 11 sites and in 2% (5/229) of the samples. Using manual search, the presence of *W*. *auropunctata* was detected at 10 additional slightly disturbed sites: 5 in Chaco/Espinal, 3 in Yungas and 2 in Paranaense Forests (S1 Table). Nests were only found in the Yungas and Chaco/Espinal Forests. In the Paranaense Forest we confirmed the presence of *W*. *auropunctata* using sieves, but we failed to find nests because baits did not attract workers, while in the other types of forests we recorded workers on only a few baits and nests were sometimes difficult to find. Most of the latter were small and contained a few workers and brood, and often the queen could not be detected.

**Fig 1:**
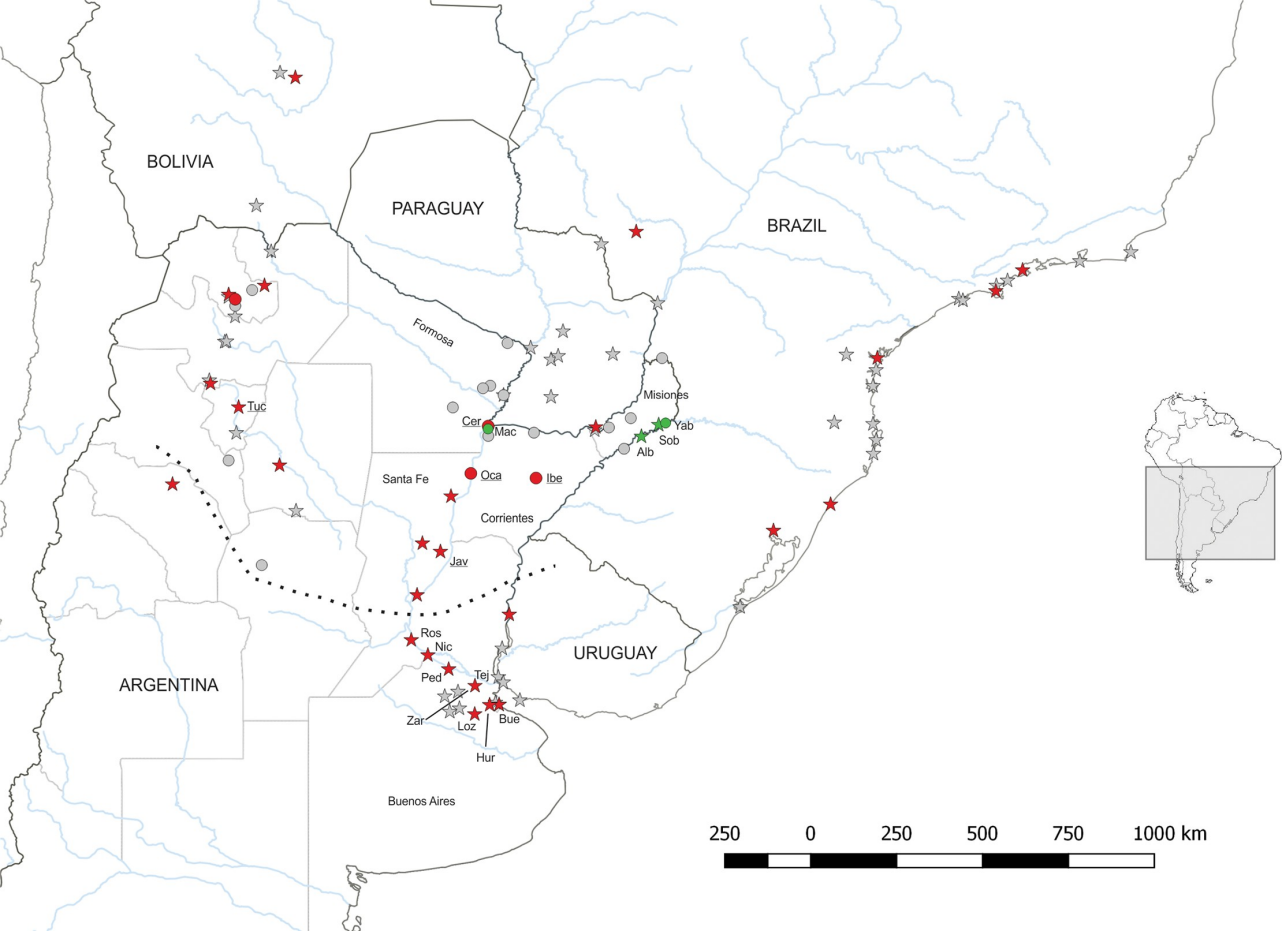
Data on the presence and reproductive system of *Wasmannia auropunctata*. The circles and stars represent natural and human-modified habitats, respectively. Symbol colour represents the type of population’s reproductive system (red: clonal, green: sexual, grey: not tested because no reproductive individuals were detected). The area below the dotted line represents the region colonised by *W*. *auropunctata* over the last 60 years. The sites mentioned in the main text are identified by the code given in S1 Table. Underlined sites specify clonal populations with more than one queen genotype.

On the contrary, in highly human-disturbed habitats, *W*. *auropunctata* was easily detected, because it usually formed conspicuous clusters composed of a variable number of nests (about 3–10), located 2–10 m apart from each other, which generally belonged to a supercolony (L.C and L.A.C unpublished data). All the populations occurring in the recently colonised area in central Argentina were established in highly human-modified habitats (Fig 1).

### Reproductive system

The multilocus genotype for each individual analysed is shown in S3 Table. Thirty-one sites were occupied by clonal populations (86%), while 4 sites by sexual populations (11%). The latter were located in northeastern Argentina: three of them (Alba Posse, El Soberbio and Yabotí) were found in the Paranaense Forest and the remaining one (camping Machuca) in a gallery forest of the Chaco/Espinal in Corrientes province (Fig 1). Three of the four sexual populations occurred in habitats with a low level of human disturbance (i.e. secondary forests or a house garden in a small tropical village), and the remainder (Yabotí) inhabited a primary forest in Misiones province. In contrast, clonal populations were mostly found in highly human-disturbed habitats like urban areas, roadsides, and a banana plantation (Yuto) in northwestern Argentina. All the sampled populations from the recently colonised area of *W*. *auropunctata* in central Argentina were clonal (Fig 1).

The clonal populations from anthropic habitats were mostly composed of one queen genotype, providing evidence that they belonged to the same clonal lineage. Interestingly, some clonal lineages from the recently colonised area spread over large distances. For instance, a same clonal lineage was found along an 83-km transect encompassing the cities of Hurlingham, Buenos Aires, and Zárate. Another genetically different clonal lineage was distributed from the floodplain of the Paraná River in Camping Las Tejas (with the highest *W*. *auropunctata* abundance) in the locality of Zárate to the City of Rosario, along a 210-km transect that included the populations from San Pedro and San Nicolás.

**Fig 2:**
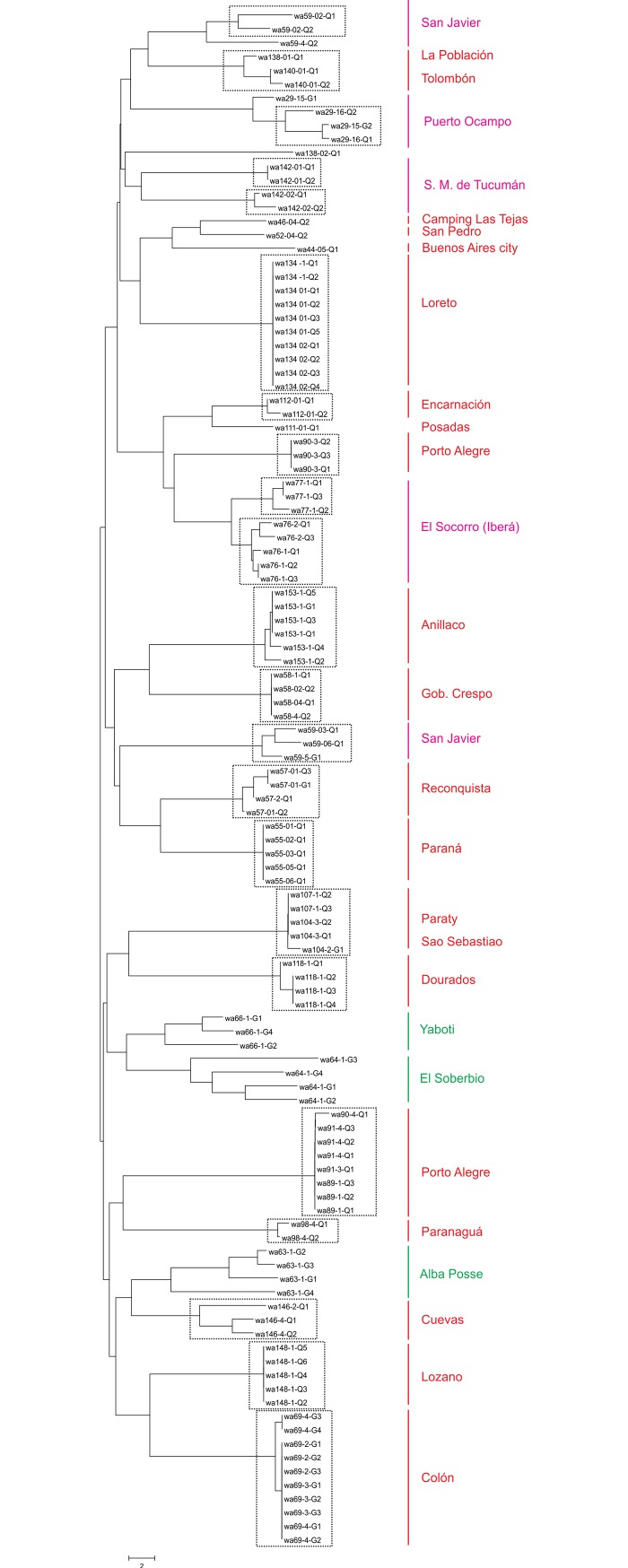
Neighbour-joining dendrogram of microsatellite distances. Each dotted line box represents a clonal lineage. The colour of each location corresponds to the type of reproductive system: green for sexual, red for clonal headed by only one queen genotype, and pink for clonal with more than one queen genotype.

It is worth mentioning that a small proportion of the clonal populations displayed more than one queen genotype. This is the case for populations from the cities of San Javier, Santa Fe province and San Miguel, Tucumán province, as well as those occurring in floodplains (i.e. Puerto Ocampo, Isla del Cerrito and Iberá) (Fig 1). In these populations, the queens of the same nest were genetically identical but different from those found in neighbouring nests, thereby revealing the existence of genetic diversity at the scale of clonal populations. The Neighbour-joining dendrogram showed that most of the different clonal lineages of the same population were closely related and constituted sister groups, suggesting common ancestry (Fig 2). The only exception was detected in San Javier, where there were three clonal lineages, one of which was genetically distant from the others (Fig 2).

## Discussion

*Wasmannia auropunctata* was much more common and abundant in highly human-disturbed habitats than in undisturbed or slightly human-disturbed habitats in its southern distribution limit in South America. As expected from previous studies [22], populations established in human-disturbed habitats were mostly clonal and exhibited ecological characteristics similar to those of invasive populations (e.g. high density of nests, workers, brood and queens). This result strengthens the tight association between human disturbance, clonality and ecological success previously reported in northern South America and within the introduced range of *W*. *auropunctata* [22]. In general, nests in human-disturbed habitats were aggregated and each aggregate (or cluster) had a single queen genotype (i.e. they were composed of a single clonal lineage).

The classical haplo-diploid sexual reproductive system was only present in four populations confined to northeastern Argentina. Three of them (Yabotí, Alba Posse and Camping Machuca) inhabited primary or secondary forests, while the fourth (El Soberbio) was found in a human-disturbed site (a house garden) surrounded by the Paranaense Forest, which is one of the best preserved forests in Argentina. Thus, there seems to be a slight association between sexual reproduction and undisturbed habitats in this region, in agreement with that reported from French Guiana [22]. In turn, sexual populations do appear to be associated with a tropical-subtropical climate with low variability in temperature and humidity, which is characteristic of northeastern Argentina. On the other hand, only clonal populations were found southwards in the provinces of Santa Fe, Entre Ríos and Buenos Aires, with a temperate climate with marked seasonality. These findings suggest a pattern of geographic parthenogenesis [43], at least within the southern limit of the native distribution of *W*. *auropunctata*, as previously documented for invasive weevils of the tribe Naupactini distributed in this same region [44], where sexual populations are in central areas and clonal populations are in marginal ones [27]. It should be kept in mind that we could not determine the reproductive system of some putative sexual populations (small nests containing very few workers in undisturbed or slightly human-modified habitats) from the Chaco/Espinal (El Bagual, San Francisco de Laishi, Chaco National Park and Pilcomayo National Park) and Yungas Forest due to the absence of breeding females or males in the nests.

All the populations from the recently colonised area (last 60 years) in central Argentina were present in human-modified habitats and (when possible) their reproductive system was assessed to be clonal. Thus, although both clonal and sexual populations are apparently distributed throughout the entire native range of *W*. *auropunctata*, only clonal populations could reach higher latitudes and colonise more extreme environments. This suggests that clonality may be associated with the southward expansion of *W*. *auropunctata* in South America. It is worth mentioning that the lack of enough sampled natural sites within the recently colonised area may represent a limitation for this conclusion because we cannot discard the presence of sexual populations in undisturbed habitats within this area. However, this sampling deficiency is because during the last 500 years central Argentina has been increasingly subjected to a variety of anthropogenic activities such as urbanisation, cattle raising and agriculture causing the disappearance of natural forests [45–47]. Nonetheless, the fact that all the populations in the expansion zone are clonal reinforces the importance of clonality as a key factor for the range expansion of this highly invasive species. Though this result was expected because all the populations were established in human-disturbed habitats, it was not obvious, since sexual populations have also been found in human-disturbed habitats in northern South America [22] and in northeastern Argentina in the present study. Furthermore, in the only sampled natural site within the recently colonised area (El Palmar Natural Reserve in Entre Ríos province) *W*. *auropunctata* was absent. In addition, surveys carried out by other authors in central Argentina also show the absence of *W*. *auropunctata* in natural ecosystems in this region [48–50]. To our knowledge, the only slightly disturbed site where *W*. *auropunctata* was recorded in this area is the Otamendi Natural Reserve, in Buenos Aires province [51]. Here, however, the species was neither abundant nor dominant and reproductive individuals were missing precluding the assessment of the reproductive system. It will be interesting to determine in the future if this population is clonal or sexual for testing the ability of *W*. *auropunctata* to colonise natural habitats during its range expansion.

Both human activity and clonality seem to play an important role in the ecological success and southern expansion of *W*. *auropunctata* in Argentina for many reasons. First, human modification of the landscape leads to the displacement of species that are unable to adapt to these changes, opening niche opportunities for tolerant species [13]. Several invasive ant species are known to be benefited in urban habitats (e.g. *Linepithema humile* [52], *Tapinoma sessile* [53] and *Lasius neglectus* [54]) and also *W*. *auropunctata* which has been described as a disturbance specialist [55]. Kusnezov was the first to propose that *W*. *auropunctata* would be the latest speciation product of the genus *Wasmannia*, extending its distribution range while displacing its natural and closely-related competitors *W*. *sulcaticeps* and *W*. *williamsoni* to marginal areas [28]. Indeed, *W*. *sulcaticeps* occurred in northern Buenos Aires 60 years ago [28] being now absent and confined to rural areas southward in Buenos Aires [50], where *W*. *auropunctata* is absent, and in the Yungas Forest, where *W*. *auropunctata* is uncommon [26]. Second, ongoing urbanisation may facilitate the southern range expansion of *W*. *auropunctata* because temperature in cities is higher than in the surrounding rural areas, a phenomenon known as the “urban heat island” (UHI) [56]. This effect has already been reported for Buenos Aires City [57], where mean temperature increased by 1°C between 1960 and 2010 [58]. Moreover, it is believed that the clonal reproductive system of *W*. *auropunctata* is selected in disturbed habitats because it enables the species to maintain over time combinations of male and female genotypes that produce workers adapted to these particular environments [22]. Taking into consideration the three aspects mentioned above, human landscape alteration would act as a vehicle for the southward spread of clonal populations of *W*. *auropunctata* in South America.

Clonality may also contribute to select genotypes that perform well in extreme environments (e.g. [59, 60]), allowing clonal populations to expand their ecological niche in relation to sexual populations. It is likely that repeated sexual reproduction events might generate allelic variants subjected to the action of natural selection, and those that are beneficial for colder climates in southern South America would become fixed by clonality. Therefore, it is reasonable to assume that clonality would have played a central role in the dispersion of *W*. *auropunctata* towards a colder climate with marked seasonality as compared to that in its native range. Indeed, workers from Zárate (central Argentina) and Israel were reported to be more tolerant to low temperatures than workers from the tropics [29]. Furthermore, the expansion that *W*. *auropunctata* experienced during the past 60 years was key for its invasion in Israel not only because it was accompanied by an adaptation to colder climates [29], but also because the species reached the surroundings of important international ports in the La Plata River basin (e.g. Rosario, Zárate and Buenos Aires). In turn, this area contains the most probable source population of Israel invasion [29].

Another factor that may explain the southward expansion of *W*. *auropunctata* in South America is linked to climate change. Empirical evidence has demonstrated that geographic distributions of many species have shifted due to changing climatic conditions [61–64]. In Argentina, a mean temperature rise of 0.5°C was registered during the last 100 years [65] and more than 100 subtropical species of plants and animals have experienced distributional shifts representing a progressive southern expansion [66]. Increases in precipitation due to climate change with the consequent increase of floods [65] may also favour the establishment of *W*. *auropunctata* southwards since this species is well adapted to floodplains [22, 30]. Indeed, the extreme discharges of the Paraná River have become considerably more frequent since the 1980s [65]. Future studies will be needed to test if the southern expansion of *W*. *auropunctata* in Argentina is, at least in part, a consequence of climate change.

It is worth mentioning that clonality may play a key role in the ecological success of *W*. *auropunctata* in human-disturbed habitats, but it would not be enough for the species to dominate natural habitats. Clonality might confer advantages to native and introduced populations conferring numerical dominance [67] which may be beneficial only in small ant assemblages (i.e. human-disturbed habitats and islands). On the contrary, in invaded natural ecosystems *W*. *auropunctata* cannot spread far beyond the limits of the forests [68, 69], being limited by biotic factors (e.g. competition). In fact, although laboratory experiments have characterised *W*. *auropunctata* as highly aggressive and dominant [70], in the wild it showed a poor performance in terms of discovery and recruitment [71] demonstrating that it is a week competitor that relies on the failure of native ants to efficiently exploit and defend food and nesting resources [72]. Thus, clonality may provide *W*. *auropunctata* with the numerical advantage for successful range expansion in anthropic environments, thereby being strongly associated with and limited to human activity, but not in natural ecosystems, where the species would not overcome biotic interactions.

Finally, it is worth noting that some clonal populations in Argentina exhibited more than one queen genotype (i.e. queens in the same nest were genetically identical but different from queens of neighbouring nests), indicating the presence of different sister clonal lineages in the same population. This pattern was observed in some populations along the Paraná River: within its floodplain (Puerto Ocampo and Isla del Cerrito) and in an urban area (San Javier). It was also found in Esteros del Iberá (El Socorro), which is a flooded environment, and in an urban area in western Argentina (San Miguel de Tucumán). Two mutually exclusive hypotheses might explain why this pattern was mostly found in flooded ecosystems. Sexual reproduction events could occasionally occur in a clonal population giving rise to new clonal lineages (as it was observed along a dirt road in French Guiana that represents a contact zone between a natural and human-modified habitat [73] and in New Caledonia [23]). On the other way around, an alternative hypothesis is that clonality may arise in an initially sexual population. Disturbance has been proposed as a force that triggers clonal or vegetative reproduction in various taxa (e.g. corals [74] and benthic invertebrates [75]). Clonality has been reported in several tree species in floodplains, which may confer greater tolerance to flooding [76]. Likewise, periodic flood pulses may promote the clonal production of several queens (and males) of *W*. *auropunctata* ensuring the survival of the colony. Because clonal reproduction requires lower energy investment and allows the production of a larger number of offspring than does sexual reproduction [67], the shift from sexual to clonal reproduction is likely to offer an immediate advantage in the short term [67] having a high probability of being positively selected. Moreover, in social insects clonality would increase colonial growth rate [77] and dispersion potential [78]. In the long term, however, parthenogenetic lineages would be condemned to extinction due to their inability to evolve [67]. Therefore, the maintenance of sexuality in populations inhabiting floodplains may be advantageous to withstand changing environmental conditions. Future studies addressing both hypotheses are necessary for in-depth understanding of the evolutionary origin of the clonal reproductive system in *W*. *auropunctata* and to test if natural (not human-derive) disturbances (e.g. periodic flood pulses in natural backwater areas) constitute driving forces for the evolution of this extraordinary reproductive system.

## Supporting information

S1 Fig Main sampling routes. Map showing the main routes along which manual search was carried out.
(TIF)

S1 Table Sampled localities. List of localities where *W*. *auropunctata* was found, geographic coordinates of each locality, brief description of the habitat, number of nests and clusters sampled per location (places where only workers were found but not nests are indicated by "workers"), number of reproductive females and males genotyped, degree of disturbance (HD: highly-disturbed, SD: slightly disturbed, UD: undisturbed) and reproductive system (C: clonal and S: sexual) of the populations. Site codes match with the ones in the map of Fig 1.
(XLSX)

S2 Table Occurrence of *Wasmannia auropunctata* in undisturbed habitats. The number of leaf-litter (sieving) and bait samples in three native forest regions of northern Argentina (Chaco/ Espinal, Yungas and Paranaense Forests) and the percentage of samples with presence of *W*. *auropunctata* are indicated. Geographic coordinates of each sampling site are informed.
(XLSX)

S3 Table Multilocus genotypes of queens (Q), gynes (G) and males (M) at 12 microsatelite loci. Sample Id. represents locality, nest and individual. Samples in bold (below the thick line) should not be analysed together with the above samples because both data sets were obtained using different ABI 3130 DNA sequencers, thus homology among alleles could not be assessed.
(XLSX)
